# Diffuse Non-Genital Cutaneous Warts

**DOI:** 10.4269/ajtmh.21-0807

**Published:** 2021-10-25

**Authors:** Ranjan Kumar Singh

**Affiliations:** Antiretroviral Therapy Centre, District Hospital, Khagaria, Bihar, India

**Figure 1.**
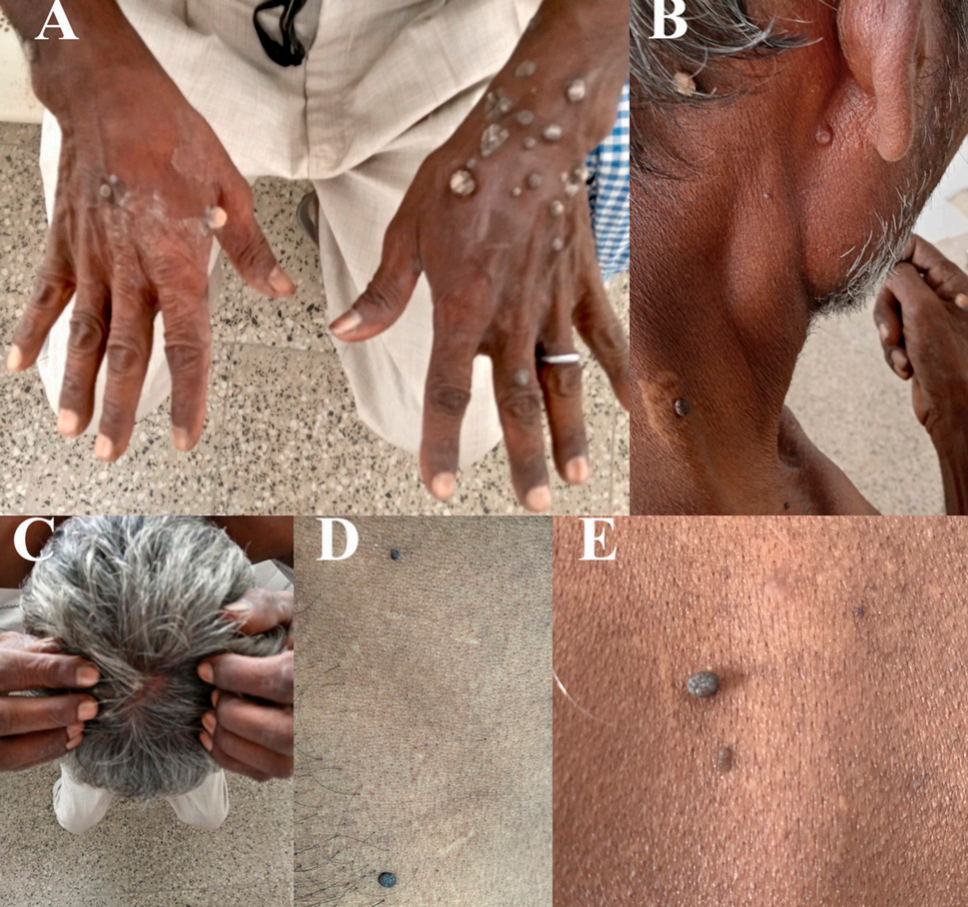
(**A–C**) Cutaneous warts with hyperkeratotic verrucous lesions. (**D, E**) Papillomatous lesions. This figure appears in color at www.ajtmh.org.

A 54-year-old man with HIV developed non-genital cutaneous warts 3 months before presentation that involved the dorsum of the hands and wrists (Figure 1A), and were sparse on the neck and scalp (Figure 1B and C). Some lesions were pedunculated papules whereas others were hyperkeratotic warts. Lesions did not resolve after topical application of salicylic acid or liquid nitrogen. New lesions continued to appear over the abdomen and back (Figure 1D and E). The patient had been taking antiretroviral (ARV) drugs—tenofovir disoproxil fumarate, lamivudine, and efavirenz—for the previous 6 years and was otherwise well. His CD4+ T-cell count was 161 cells/µL and his HIV-1 viral load was 555,221 copies/mL despite good adherence to ARV drugs. These parameters indicated the patient’s current ARV drugs were losing efficacy. His ARV drug regimen was changed to abacavir, lamivudine, and dolutegravir. Thereafter, the patient experienced no new lesions on his body. The existing warty lesions became black, and a number of them fell off. The patient had HIV-1 suppression (viral load, 68 copies/mL) 3 months after changing the ARV drug regimen.

Cutaneous warts are usually self-limited lesions, with a greater prevalence in HIV-positive individuals.^1^ The diagnosis is based on clinical appearance. A subset of human papilloma virus (HPV) types with tropism for cutaneous epithelial cells causes non-genital warts. Immunosuppression may trigger latent HPV reactivation.^2^ In our patient, widespread warts appeared either as a result of the reactivation of HPV or de novo infection after progressive HIV-1 immunosuppression related to drug resistance. Diffuse warts in an HIV-positive individual may indicate decreased efficacy of the current ARV drug regimen.
